# Advancing the accuracy of SARS-CoV-2 phosphorylation site detection via meta-learning approach

**DOI:** 10.1093/bib/bbad433

**Published:** 2023-12-06

**Authors:** Nhat Truong Pham, Le Thi Phan, Jimin Seo, Yeonwoo Kim, Minkyung Song, Sukchan Lee, Young-Jun Jeon, Balachandran Manavalan

**Affiliations:** Department of Integrative Biotechnology and of Biopharmaceutical Convergence, Sungkyunkwan University, Suwon 16419, Gyeonggi-do, Republic of Korea; Department of Integrative Biotechnology and of Biopharmaceutical Convergence, Sungkyunkwan University, Suwon 16419, Gyeonggi-do, Republic of Korea; Department of Integrative Biotechnology and of Biopharmaceutical Convergence, Sungkyunkwan University, Suwon 16419, Gyeonggi-do, Republic of Korea; Department of Integrative Biotechnology and of Biopharmaceutical Convergence, Sungkyunkwan University, Suwon 16419, Gyeonggi-do, Republic of Korea; Department of Integrative Biotechnology and of Biopharmaceutical Convergence, Sungkyunkwan University, Suwon 16419, Gyeonggi-do, Republic of Korea; Department of Integrative Biotechnology and of Biopharmaceutical Convergence, Sungkyunkwan University, Suwon 16419, Gyeonggi-do, Republic of Korea; Department of Integrative Biotechnology and of Biopharmaceutical Convergence, Sungkyunkwan University, Suwon 16419, Gyeonggi-do, Republic of Korea; Department of Integrative Biotechnology and of Biopharmaceutical Convergence, Sungkyunkwan University, Suwon 16419, Gyeonggi-do, Republic of Korea

**Keywords:** bioinformatics, meta-learning approach, phosphorylation sites, feature selection, machine learning

## Abstract

The worldwide appearance of severe acute respiratory syndrome coronavirus 2 (SARS-CoV-2) has generated significant concern and posed a considerable challenge to global health. Phosphorylation is a common post-translational modification that affects many vital cellular functions and is closely associated with SARS-CoV-2 infection. Precise identification of phosphorylation sites could provide more in-depth insight into the processes underlying SARS-CoV-2 infection and help alleviate the continuing COVID-19 crisis. Currently, available computational tools for predicting these sites lack accuracy and effectiveness. In this study, we designed an innovative meta-learning model, Meta-Learning for Serine/Threonine Phosphorylation (MeL-STPhos), to precisely identify protein phosphorylation sites. We initially performed a comprehensive assessment of 29 unique sequence-derived features, establishing prediction models for each using 14 renowned machine learning methods, ranging from traditional classifiers to advanced deep learning algorithms. We then selected the most effective model for each feature by integrating the predicted values. Rigorous feature selection strategies were employed to identify the optimal base models and classifier(s) for each cell-specific dataset. To the best of our knowledge, this is the first study to report two cell-specific models and a generic model for phosphorylation site prediction by utilizing an extensive range of sequence-derived features and machine learning algorithms. Extensive cross-validation and independent testing revealed that MeL-STPhos surpasses existing state-of-the-art tools for phosphorylation site prediction. We also developed a publicly accessible platform at https://balalab-skku.org/MeL-STPhos. We believe that MeL-STPhos will serve as a valuable tool for accelerating the discovery of serine/threonine phosphorylation sites and elucidating their role in post-translational regulation.

## INTRODUCTION

The onset of the severe acute respiratory syndrome coronavirus 2 (SARS-CoV-2) in 2019 sparked a global pandemic and posed significant challenges to human health [1]. Elucidating the mechanisms underlying SARS-CoV-2 infection is vital for devising efficacious treatments against COVID-19. Infection disrupts signaling cascades, leading to protein–protein interactions between human and viral components [2, 3]. SARS-CoV-2 instigates host kinase activation, augmenting phosphorylation rates in both viruses and host organisms. Recent investigations have revealed approximately 70 phosphorylation sites in SARS-CoV-2 proteins, alongside over 15 000 host phosphorylation sites in infected cells [4]. In addition, human missense single-nucleotide variants that alter phosphorylation sites during SARS-CoV-2 infection have been identified, contributing to inter-individual variability in infection susceptibility and subsequent pneumonia manifestation [5]. Phosphorylation, a post-translational modification, modulates cellular processes and protein functions [6], with the phosphorylation state of the SARS-CoV-2 nucleocapsid protein affecting its binding affinity to the host 14-3-3 protein [7, 8]. Phosphatase has emerged as a promising drug target against SARS-CoV-2 [9], suggesting that addressing how phosphorylation is regulated within the virus may prove instrumental in combating the persistent COVID-19 pandemic.

Advances in biotechnology have increased the availability of high-throughput sequencing techniques [10]. These methods are proficient at identifying phosphorylation sites, though they can be expensive and time intensive. The extensive phosphorylation data produced using these techniques offer valuable resources for developing machine learning (ML)-based computational models to identify phosphorylation sites. Gao *et al*. presented Musite [11], an approach based on support vector machine (SVM) that employs protein disorder predictors, amino acid frequencies and *k*-nearest neighbor scores. Dou *et al*. established PhosphoSVM [12], which integrates eight unique features. RF-Phos [13] implemented a random forest (RF) algorithm that merges structural characteristics and sequence information as input features. PhosphoPredict [14], proposed by Song *et al*., is an RF-oriented method that uses heterogeneous features. However, the performance of these ML-driven approaches relies heavily on feature quality. Although various feature types and feature selection techniques have been employed to improve model efficacy, the generalizability of these methods remains suboptimal.

The surge in high-performance hardware capabilities and the abundance of data have encouraged the implementation of deep learning techniques for phosphorylation site identification. MusiteDeep2017 [15] employs a convolutional neural network (CNN) to detect kinase-specific phosphorylation sites. DeepPhos [16], on the other hand, utilizes CNNs to recognize both general and kinase-specific phosphorylation sites, surpassing existing predictors in performance. MusiteDeep2020 [17] identifies phosphorylated serine (S)/threonine (T) and tyrosine (Y) sites using a Capsule Network (CapsNet). More recently, Lv and colleagues introduced DeepIPs [18], which combines long short-term memory (LSTM) and a CNN to predict phosphorylated S/T and Y sites. Notably, DeepIPs stands alone among the relevant prediction tools for its ability to detect phosphorylation sites in SARS-CoV-2-infected cells. Despite considerable advancements and its popularity within the research community, the method presents certain shortcomings: (i) the use of word-embedding vectors neglects the amino acid composition, physicochemical properties and position-specific information of the protein; (ii) the deep learning model was constructed without systematically assessing conventional classifiers; (iii) DeepIPs considered only A549 cells and did not consider other cell types; (iv) there is room for further improvement and optimization of the current method.

**Figure 1:**
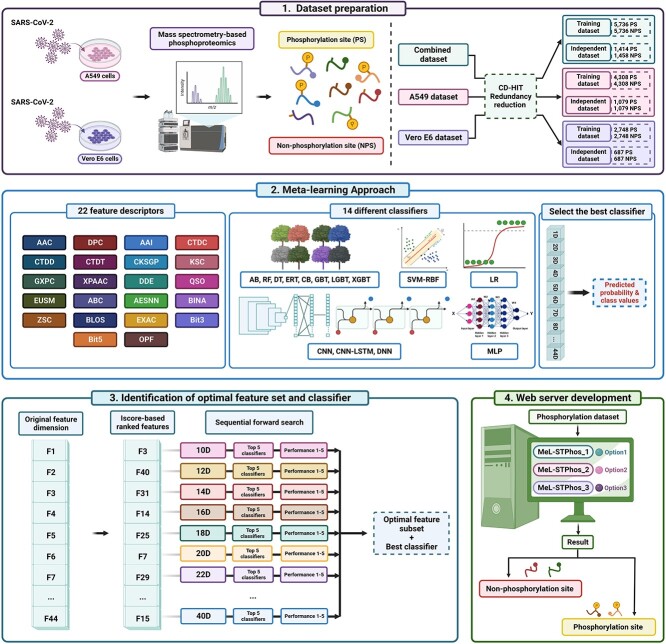
The overall framework of Meta-Learning for Serine/Threonine Phosphorylation (MeL-STPhos) construction (created with BioRender.com).

Here, we initially established that SARS-CoV-2 infection induces alterations in phosphorylation, as evidenced by bioinformatics analysis of A549 cells infected with the virus. We subsequently proposed a novel SARS-CoV-2 phosphorylation modification site predictor (MeL-STPhos) using a meta-learning approach (Figure 1). In this study, we constructed three different S/T datasets, including A549 cells, Vero E6 cells, and a combination of A549 and Vero E6 cells. For each dataset, we investigated 29 feature-encoding algorithms that encompass various aspects of sequence information and 14 different classifiers, including conventional and deep learning algorithms. We selected the most effective model for each feature, integrated predictions and identified the most critical base models through a rigorous feature selection strategy. To the best of our knowledge, this is the first study to report the two cell-specific models and a generic model for phosphorylation site prediction by exploiting extensive range of sequence-derived features and ML algorithms. Furthermore, we have conducted the case study to identify S/T phosphorylation sites in lung fibroblast cells (IMR-90 infected with adenovirus type 2). Comprehensive comparative experiments revealed that MeL-STPhos surpasses current state-of-the-art methodologies, indicating the enhanced effectiveness and potential of meta-learning approaches compared to word-embedding features. Furthermore, we provide MeL-STPhos as a web server, facilitating the exploration of novel phosphorylation modification sites in SARS-CoV-2 infection and contributing to an improved understanding of associated pathogenesis and therapeutic strategies.

## MATERIALS AND METHODS

### Methods for bioinformatics analysis

#### Differentially expressed gene analysis

The mRNA-sequencing (mRNA-seq) dataset GSE184536 in Gene Expression Omnibus (GEO; https://www.ncbi.nlm.nih.gov/geo/) was downloaded as a raw-counts table. It contains gene expression data from the angiotensin-converting enzyme 2-expressing A549 cell line infected with SARS-CoV-2 and uninfected cell line samples over time (4, 6, 9 and 24 h post-infection (hpi)). The datasets of 2 and 12 hpi were excluded because of the sequencing quality and loss of triplicates, respectively (refer to supplementary information). The raw expression files were divided into four groups by hours and they were each normalized using the DESeq2 package (v.1.36.0) [19]. Before normalization, the raw count data were filtered based on the filtering criteria of expression in at least two-thirds of the samples for each group. The cut-off value for DEGs was |log2 fold change| >1.5 and adjusted *P*-value <0.01. In addition, in this study we used principal component analysis (PCA), weighted correlation network analysis (WGCNA), Gene Ontology (GO) and single-sample Gene Enrichment Analysis (ssGSEA). Comprehensive details of these procedures are provided in the supplementary information.

#### Validation of DEG analysis in vivo data

To confirm the analysis of DEGs in SARS-CoV-2 infected cell line data, we selected a publicly available mRNA-seq dataset (GSE190496) and downloaded it from the GEO database. The dataset contains gene expression data from five formalin-fixed paraffin-embedded (FFPE) normal human lung samples and 12 FFPE COVID-19 patient lung tissue samples. We first grouped the raw expression dataset by COVID-19 infection status and normalized it using the DESeq2 package. Before normalization, we filtered the raw count data to remove genes that were not expressed in at least two-thirds of the samples for each group. The cut-off value for DEGs was |log2 fold change| >1.5 and adjusted *P*-value <0.01.

### Data collection

We constructed three different datasets: (i) we employed the same dataset as that used in DeepIPs [18], which contains 14 119 experimentally verified phosphorylation sites from human A549 cells infected with SARS-CoV-2 [20]. To create a high-quality dataset, CD-HIT [21] was used to eliminate redundant sequences by applying a sequence similarity threshold of 30%. The processed sequences were then converted into 33-residue segments with S/T. Segments with phosphorylated central S/T were labeled positive, while the others were considered negative samples. This approach generated a considerable number of negative samples. To prevent bias and overfitting during model training, non-redundant negative samples were randomly selected to match the number of positive samples. The final training samples for the development of the prediction model consisted of 4308 positive and 4308 negative samples. To assess the transferability of the developed model, an independent dataset was created that contained 1079 positive and 1079 negative samples. This dataset is named as A549 dataset. (ii) We extracted phosphorylation data from Vero E6 cells infected with SARS-CoV-2 [22] and applied CD-HIT threshold of 30%, which resulted in 3435 positive and 191 008 negative samples. As mentioned above, we randomly selected the same number of negative samples as positives. From the total samples, we randomly selected 2748 positive and 2748 negative samples for the model training, and the remaining samples (687 positive samples and 687 negative samples) for the model validation. This dataset is named as Vero E6 dataset. (iii) The combined dataset was generated by merging A549 and Vero E6 datasets. The training samples of both datasets were combined, and redundancy was excluded by applying CD-HIT with a threshold of 80%. This resulted in 5736 and 5736 negative samples for model development. Similarly, two independent datasets were combined and samples that overlapped with the training dataset were excluded, which resulted in 1414 positive and 1458 negative samples for independent assessment.

### Feature encodings

In our study, we used 29 distinct feature encodings to represent various sequence attributes, based on the assumption that these encodings possess significant discriminative power for distinguishing between positive and negative samples. Some feature encodings were merged. For example, the grouped dipeptide and tripeptide compositions were combined, resulting in ‘GXPC’. Consequently, we derived the following 22 feature descriptors (from the initial 29 feature encodings) using various packages [23–26]: amino acid composition (AAC), dipeptide composition (DPC), amino acid index (AAI), composition-transition-distribution (CTDC, CTDT, CTDD), composition of *k*-spaced amino acid group pairs (CKSGP), *k*-spaced conjoint triad (KSC), GXPC, XPAAC (a linear combination of pseudo AAC (PAAC) and amphiphilic PAAC), dipeptide deviation from the expected mean (DDE), quasi-sequence-order (QSO), EGSM (a combination of Shannon **E**ntropy, **G**eary autocorrelation, **S**equence-order coupling number and **M**oran autocorrelation), atomic and bond compositions (ABC), AESNN (derived from alignments), binary 3 bit (Bit3), binary 5 bit (Bit5), overlapping property features (OPF), binary profile (BINA), Z-scale (ZSC), BLOSUM62 (BLOS), and a linear combination of enhanced AAC and enhanced grouped AAC (EXAC). A concise overview of these encodings is presented in the supplementary information.

### Meta-learning approach

The meta-learning approach consisted of three stages: (i) building the baseline models; (ii) choosing the best-performing model for each feature descriptor; (iii) identifying the ideal baseline models in conjunction with the most suitable classifier.

#### Construction of baseline models

The 22 feature descriptors were individually input into the following 14 classifiers: RF, CNN, deep neural network (DNN), CNN-LSTM, extremely randomized trees (ERT), multi-layer perceptron (MLP), SVM, gradient boosting trees (GBT), extreme GBT (XGBT), AdaBoost (AB), catBoost (CB), light GBT (LGBT), decision trees (DT) and logistic regression (LR). During model training, we used a statistical technique called 10-randomized 10-fold cross-validation (CV). This method involves randomly dividing the data into 10 parts and repeating the process 10 times. We calculated the average performance metrics for each model across all 10 iterations. In comparison to a single 10-fold CV, this strategy provides several advantages, such as decreased bias, more reliable performance estimations, enhanced resilience to outliers and superior model selection [27]. We utilized grid search to optimize each classifier’s hyperparameters during training, with a specified range based on previous studies [28–34]. For implementing a 10-randomized 10-fold CV, the median parameter from the 10 sets was selected to establish the final baseline model. In total, we generated 308 baseline models (22 feature descriptors multiplied by 14 classifiers) for each dataset. Model performances were assessed using standard evaluation metrics, such as sensitivity (Sn), specificity (Sp), AUC (area under the receiver operating characteristic (ROC) curve), accuracy (ACC) and Matthews correlation coefficient (MCC). The conventional definitions of these metrics can be found in other sources [35, 36].

#### Selection of the best model for each descriptor

Previous studies have examined several models for each descriptor and employed them to create meta-predictors [31, 37]. However, employing numerous ML-based models for each descriptor may lead to the incorporation of redundant or biased information during the meta-learning process. Consequently, we selected the best-performing model from the 14 distinct classifiers available for each descriptor. Ultimately, we acquired the 22 optimal baseline models for each dataset based on various ML classifiers.

#### Identifying the ideal baseline models in conjunction with the most suitable classifier

The estimated probabilities of phosphorylation sites (i.e. positive instances) and class labels derived from the 22 baseline models were combined to generate 44-dimensional (44-D) feature vectors. Considering that not all 44-D features hold equal significance and some may be irrelevant, we implemented a two-stage feature selection approach [38] to identify the most pertinent features within each dataset. This method merges feature scoring functions with sequential forward search (SFS), providing an equilibrium between computational efficiency and model precision. In general, tree-based classification algorithms can inherently rank features according to their feature importance scores (FIS). However, these scores alone may not provide sufficient accuracy in ranking features from the most to least important. To address this issue, we propose a method for selecting optimal models using 44-D features across six tree-based classifiers (AB, RF, ERT, XGBT, LGBT and CB). Each classifier independently calculates FIS. Given that certain classifiers (XGBT and LGBT) yield relative scores outside the 0–1 range, we normalized FIS values for all six classifiers and computed the average score for each feature. We termed this aggregated score as Iscore.

Based on Iscore values, we used SFS to iteratively construct feature subsets by including the top two ranked features at each iteration [39]. This process generated 16 distinct feature subsets ranging from 10- to 40-D features. These subsets were subsequently fed into the top five classifiers, which were selected based on their average performance across the 22 feature descriptors. The performances of these models were compared to identify the best-performing classifier and the corresponding feature dimension was considered optimal. This methodology is applicable to a broad array of ML problems and is particularly useful when handling high-dimensional datasets.

## RESULTS AND DISCUSSION

### Unraveling the transcriptomic response of A549 cells to SARS-CoV-2 infection through bioinformatics analysis

**Figure 2:**
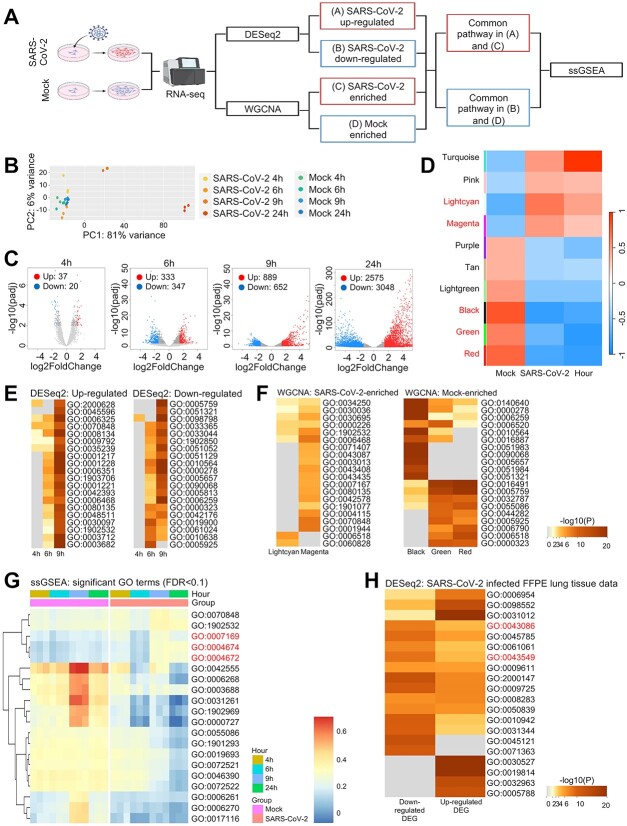
(**A**) The schematic diagram shows the methods for RNA-sequencing (RNA-seq) data analysis. The raw data (GSE184536) were subject to two analytical packages, DESeq2 and weighted correlation network analysis (WGCNA). The identified gene sets were functionally annotated using Gene Ontology (GO) analysis, and the characterized groups were assessed using single-sample Gene Enrichment Analysis (ssGSEA). (**B**) Principal component analysis (PCA) using the top 500 genes with the largest variance across A549 cells infected with severe acute respiratory syndrome coronavirus 2 (SARS-CoV-2) and uninfected cells at different hours post-infection. (**C**) Volcano plots indicating differentially expressed genes (DEGs) from the RNA-seq analysis of A549 cells infected with SARS-CoV-2 compared to uninfected cells. Red dots indicate genes with a log2(fold change) >1.5 and an adjusted *P*-value of <0.01. Blue dots indicate genes with a log2(fold change) <−1.5 and an adjusted *P*-value of <0.01. Genes with no significantly different expression are shown in gray. (**D**) WGCNA cluster–trait relationships (SARS-CoV-2-infected versus mock cells by hours). Each row corresponds to a co-expressed gene cluster and columns, to trait data. Each cell is color coded based on correlation statistics according to the color legend on the right. Row labels in bold red are mock- and SARS-CoV-2-related significant clusters selected by *P*-value (‘Turquoise’ was a significant cluster but not enriched in GO terms; it was thus not selected). (**E**, **F**) Heatmaps of Metascape enrichment analyses across dysregulated genes in SARS-CoV-2-infected cells. Genes with upregulated or downregulated expression are shown in the left and right panels, respectively (**E**). WGCNA clusters enriched in mock and SARS-CoV-2-infected cells are shown in the left and right panels, respectively (**F**). The top 20 significant GO terms are shown. (**G**) Heatmap showing the ssGSEA scores of the top 5 gene sets with increased and top 15 with decreased expression (from **E** and **F**, respectively). The top 5 significant pathways were observed in both upregulated DEGs and SARS-CoV-2-related clusters, and the remaining 15 pathways were significant in both downregulated DEGs and mock-related clusters (FDR <0.1 using Wilcoxon rank-sum test comparing mock and SARS-CoV-2-infected cells). The GO terms, specifically ‘GO:0007169’, ‘GO:0004674’ and ‘GO:0004672’, marked in red, represent ‘transmembrane receptor protein tyrosine kinase signaling pathway’, ‘Protein serine/threonine kinase activity’ and ‘Protein kinase activity’. (**H**) Heatmaps of Metascape enrichment analyses across dysregulated genes in lung tissues infected with SARS-CoV-2. The top 20 significant GO terms are shown and the red marked GO terms (GO:0043086 and GO:0043549) indicate ‘Regulation of kinase activity’ and ‘Negative regulation of catalytic activity’, respectively.

To investigate transcriptomic signatures in response to COVID-19 infection, we used A549 cells infected with SARS-CoV-2 (GSE184536) and analyzed the signatures using two methods: DESeq2-based gene expression and WGCNA-based cluster identification (Figure 2A). First, we analyzed DEGs using the DESeq2 package and subsequent PCA using the 500 genes with the largest variance. The results showed that SARS-CoV-2 infection dramatically changed the transcriptomes at 9 and 24 hpi compared to the mock and asymptomatic periods (Figure 2B and C). These data are consistent with a previous observation that viremia is observed from 9 hpi [40] and is associated with significantly increased dysregulation of gene expression after infection.

Next, we used WGCNA to identify clusters of dysregulated genes associated with SARS-CoV-2 infection and detected five significantly enriched clusters (Figure 2D). We then performed GO analysis to functionally characterize both the DEGs and clusters and displayed the top 20 GO terms for each (Figure 2E and F). Overall, 255 overlapping GO terms were identified in both analyses. The gene sets in the most significant top 15 GO terms related to mock infection and 5 terms related to SARS-CoV-2 infection were analyzed in individual samples using ssGSEA. ssGSEA revealed that protein phosphorylation pathways were significantly enriched at 9 and 24 hpi, whereas the GO terms of DNA replication and DNA/RNA synthesis were significantly enriched in mock-treated A549 cells (Figure 2G).

To further validate our findings, we employed 5 normal and 12 infected lung tissue samples and performed transcriptome-wide analysis (GSE190496). PCA showed that the infected samples were clearly separated from the normal tissue samples (Figure S1A available online at http://bib.oxfordjournals.org/). DESeq-2 identified around 1000 genes that were differentially expressed between two samples. Functional annotation using the Metascape package revealed that the genes were associated with the regulation of kinase activity and the negative regulation of catalytic activity (Figure S1B available online at http://bib.oxfordjournals.org/ and Figure 2H). These results are consistent with the findings from A549 cell lines, which further support our conclusion that protein phosphorylation events are significantly altered upon cellular infection with SARS-CoV-2.

### Exploring phosphorylation site prediction through extensive ML analysis on different datasets

**Figure 3:**
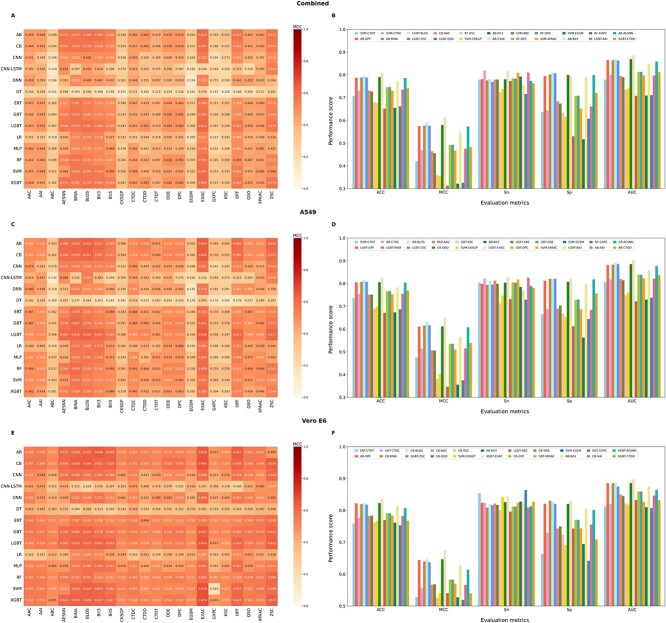
Comparison of performance among various baseline models and identification of the best classifier for each descriptor. The comparison based on Matthews correlation coefficient (MCC) scores of 308 baseline models for each of the combined, A549 and Vero E6 datasets is displayed in panels (**A**), (**C**) and (**E**), respectively. The chosen best classifier for each descriptor, totaling 22 models for the combined, A549 and Vero E6 datasets, is depicted in panels (**B**), (**D**) and (**F**), respectively.

We used 22 feature descriptors that encompass a comprehensive range of properties derived from sequencing data. We evaluated their discriminative capabilities in predicting phosphorylation sites using 14 distinct classifiers, including both shallow and deep learning algorithms. For details on the feature descriptors and classifiers, please refer to the Methods section. We classified descriptors as having high, moderate or low discriminative capacities based on MCC values of >0.600, 0.400–0.600 and <0.400, respectively, by at least one classifier. When we applied this criterion to the combined dataset (Figure 3A), we observed that EXAC descriptor had high discriminative capacity, five descriptors (ABC, CKSGP, EGSM, GXPC and KSC) with low discriminative capacity, and the rest had moderate discriminative capacity. For the A549 dataset, we identified seven descriptors (AESNN, BINA, BLOS, Bit3, OPF, EXAC and ZSC) with high discriminative capacity, four descriptors (EGSM, ABC, GXPC and KSC) with low discriminative capacity, while the remaining descriptors had moderate discriminative capacity (Figure 3C). Finally, when we applied this criterion to the Vero E6 dataset, we observed that the top eight descriptors (AESNN, BINA, BLOS, Bit3, Bit5, EXAC, ZSC and OPF) exhibited high discriminative capacity, while the remaining descriptors showed moderate discriminative capacity, and there was no descriptor had low discriminative capacity (Figure 3E).

Next, we compared the performances of 14 different classifiers with respect to each descriptor to determine the best model. Notably, we observed a similar tendency in all three datasets, where some classifiers demonstrated the best performance on different descriptors, while most of the classifiers did not perform best on any single descriptors out of 22 descriptors. For the combined dataset, AB achieved the best performance on six descriptors (OPF, BINA, Bit3, EXAC, Bit5 and AESNN), SVM with six other descriptors (CTDT, CTDC, CKSGP, ABC, XPAAC and EGSM), LGBT with four descriptors (BLOS, ZSC, QSO and AAI), RF with four different descriptors (KSC, DPC, DDE and GXPC), and CB and XGBT respectively with AAC and CTDD descriptors (Figure 3B). Notably, the AB-EXAC model achieved the highest performance among the 22 best baseline models, with the MCC of 0.613, ACC of 0.806 and AUC of 0.886. More specifically, its MCC value was 2.09 to 30.08% higher than those of the other models. In the case of the A549 dataset, SVM, LGBT, AB, GBT, CB, MLP and RF achieved their best performance on four (CTDT, CKSGP, XPAAC and EGSM), six (BINA, ZSC, EXAC, ABC, Bit5 and OPF), five (CTDC, BLOS, Bit3, AAI and CTDD), three (KSC, DPC and DDE), two (QSO and AESNN), AAC and GXPC descriptors, respectively (Figure 3D). Of the 22 best baseline models for each descriptor, the LGBT model based on EXAC demonstrated the highest ACC, MCC and AUC values at 0.649, 0.825 and 0.902, respectively. Notably, the MCC value was 1.74–30.33% greater than those of the other models based on different descriptors. Interestingly, the ACC score of the LGBT-EXAC model was 2.00% higher than that of the existing best predictor, DeepIPs, which was developed on the same training dataset. In the case of the Vero E6 dataset, CB outperformed other classifiers on eight descriptors (BINA, BLOS, AAC, QSO, KSC, DPC, DDE and AAI), XGBT excelled on four descriptors (ZSC, EXAC, AESNN and CTDD), AB was more effective on three descriptors (OPF, Bit3 and Bit5), GBT dominated on three descriptors (CTDC, XPAAC and GXPC), SVM showed its potential on two descriptors (CKSGP and EGSM), and LGBT and ERT were the best on ABC and CTDT descriptors, respectively (Figure 3F). It is worth noting that the XGBT-EXAC model emerged as the best baseline model, achieving the highest MCC of 0.674, ACC of 0.837 and AUC of 0.898. This model’s MCC outperformed other 21 descriptor-based classifiers by 2.79–15.55%, highlighting its relative effectiveness.

Overall, we found that these 14 classifiers performed differentially on different datasets and feature descriptors. Specifically, six classifiers (AB, SVM, LGBT, RF, CB and XGBT) performed well on the combined dataset, seven classifiers (SVM, LGBT, AB, GBT, CB, MLP and RF) performed well on the A549 dataset, and a slightly different set of seven classifiers (CB, XGBT, AB, GBT, SVM, LGBT and ERT) performed well on the Vero E6 dataset. Notably, tree-based classifiers contributed significantly to the top-performing models regardless of datasets. Although most existing methods rely on deep learning-based algorithms [18, 41] and often use word-embedding vectors as features, our study is the first examination of such an extensive range of feature descriptors and classifiers for phosphorylation site prediction. We assessed the discriminative ability of each feature descriptor and the capacity of the classifiers at predicting on different datasets, laying the groundwork for future studies when larger datasets become available. To enhance the performance of the best baseline models, we next considered all the 22 best baseline models and investigated a meta-learning approach.

### Development of MeL-STPhos

Typically, baseline models provide two distinct types of information: predicted probability scores and class labels. Using the 22 optimal models, we obtained 22-D probabilistic feature vectors (PFV) and class label feature vectors (CFV). In addition, we generated 44-D feature vectors (PCFV) by integrating PFV and CFV. To develop a prediction model using this information, the most suitable classifiers for predicting phosphorylation sites must be identified. We focused on the top five classifiers rather than the 14 classifiers used for constructing the baseline models. To select the top five classifiers for each dataset, we averaged the performance of each classifier for the 22 descriptors and ranked them based on their MCC values. Figure S2 available online at http://bib.oxfordjournals.org/ shows that the top five classifiers for the combined dataset were RF, XGBT, AB, LGBT and CB. The top five classifiers for the A549 dataset were AB, LGBT, CB, GBT and RF. Also, the top five classifiers for the Vero E6 dataset were AB, XGBT, GBT, LGBT and CB. Using these classifiers, we developed prediction models based on PFV, CFV and PCFV.

**Figure 4:**
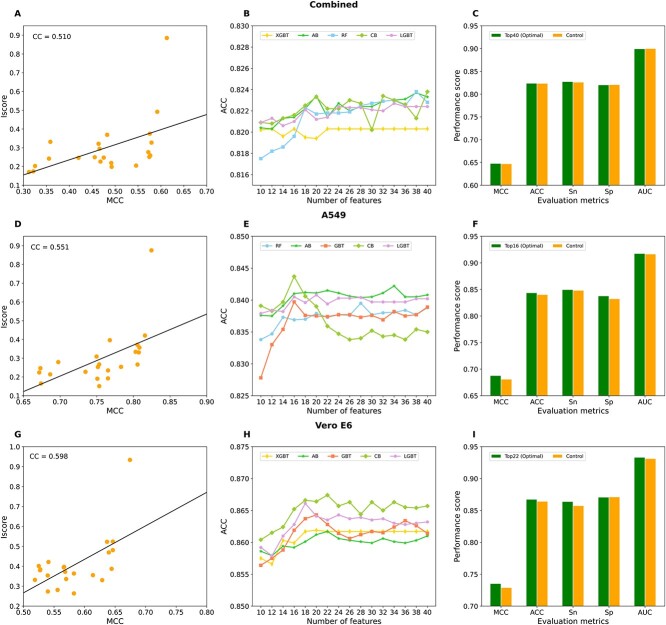
The correlation coefficient between the Iscore and the corresponding MCC values of each feature for the combined (**A**), A549 (**D**) and Vero E6 (**G**) datasets. The performances of five classifiers using different feature subsets are displayed for the combined (**B**), A549 (**E**) and Vero E6 (**H**) datasets; these subsets were generated based on Iscore. Performance comparison between the optimal model and control for the combined (**C**), A549 (**F**) and Vero E6 (**I**) datasets.

Figure S3 available online at http://bib.oxfordjournals.org/ demonstrates that the classifiers based on PCFV performed marginally better than those using PFV and CFV, regardless of the dataset. Consequently, we implemented a feature selection protocol using PCFV (Methods section). We first computed the Iscore and compared it to the actual baseline MCC to understand their relationship. This yielded a correlation coefficient (CC) of 0.510 for the combined dataset, 0.551 for the A549 dataset and 0.598 for the Vero E6 dataset, indicating a moderate relationship (Figure 4A, D and G). In other words, the highest MCC of the baseline models does not necessarily correspond to a high Iscore. By leveraging different feature subsets, we generated five distinct classifier models for each subset and compared their performance. The results indicated that CB with 40-D features achieved the highest ACC of 0.824 on the combined dataset, which was a slight improvement compared to the control (Figure 4C). On the A549 and Vero E6 datasets, CB classifiers with 16-D and 22-D features achieved ACCs of 0.844 and 0.867, respectively, a notable improvement compared to the control (Figure 4F and I). These three models have been designated as the final models for predicting phosphorylation sites across the three datasets and are named MeL-STPhos. Notably, MeL-STPhos_1, MeL-STPhos_2 and MeL-STPhos_3 were specifically designed for the combined, A549 and Vero E6 datasets, respectively. Interestingly, the CB classifier ranked fourth and third for the combined and A549 datasets, respectively, but was the top performer for the Vero E6 dataset (Figure S2 available online at http://bib.oxfordjournals.org/). When we built a meta-learning model using baseline information, CB outperformed the other classifiers on all three datasets. This highlights the benefits of investigating the performance of multiple classifiers rather than focusing on a single one.

### Evaluating MeL-STPhos against leading baseline models on the same training and independent datasets

**Figure 5:**
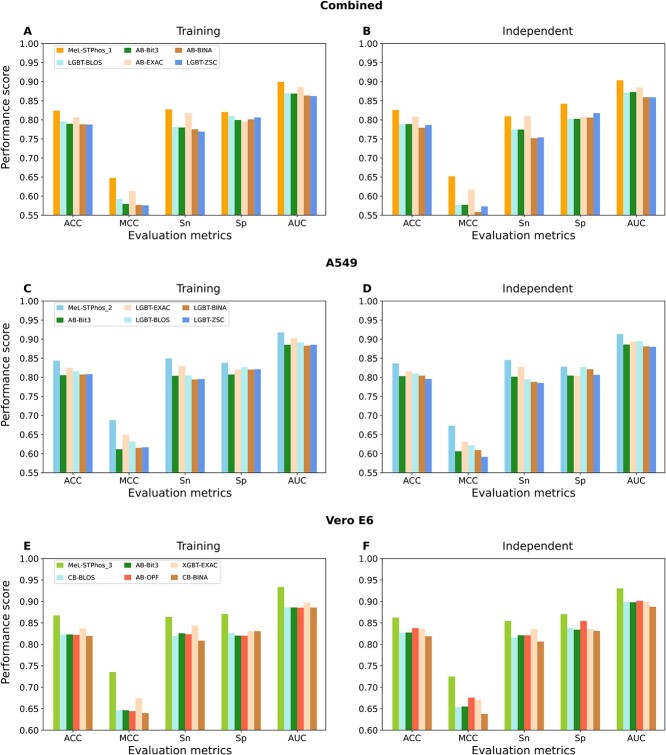
Comparison of performance between MeL-STPhos and the top baseline models using training and independent datasets. Performance on the combined training (**A**) and combined independent datasets (**B**). The corresponding performance on the A549 dataset is shown in (**C**) and (**D**), respectively. Performance on the Vero E6 training (**E**) and Vero E6 independent datasets (**F**).

On the combined dataset, MeL-STPhos_1 outperformed the top five baseline models, achieving ACC, MCC, Sn, Sp and AUC of 0.824, 0.648, 0.827, 0.820 and 0.899, respectively (Figure 5A). These metrics represent improvements of 1.75–3.61%, 3.49–7.20% and 1.37–3.72% over the top five baseline models (AB-EXAC, LGBT-BLOS, AB-Bit3, AB-BINA and LGBT-ZSC). In the A549 dataset, MeL-STPhos_2 outperformed the top five baseline models (LGBT-EXAC, AB-BLOS, LGBT-ZSC, LGBT-BINA and AB-Bit3) by 1.91–3.81% in ACC, 3.84–7.68% in MCC and 1.54–3.23% in AUC (Figure 5C). Notably, MeL-STPhos_2 achieved the MCC, ACC, Sn, Sp and AUC values of 0.688, 0.844, 0.850, 0.838 and 0.918, respectively. Similarly, MeL-STPhos_3 significantly outperformed the top five baseline models on the Vero E6 dataset (Figure 5E). It reached the highest MCC, ACC, Sn, Sp and AUC values of 0.735, 0.867, 0.864, 0.871 and 0.933, respectively, which were 6.10–9.56% higher in MCC, 3.04–4.79% higher in ACC and 3.58–4.76% higher in AUC than the top five baseline models (CB-BLOS, AB-Bit3, AB-OPF, XGBT-EXAC, CB-BINA).

To assess the robustness of MeL-STPhos, we tested its performance on their respective independent datasets and compared these performances with the top five baseline models. Figure 5 shows that MeL-STPhos models consistently outperformed the top five baseline models on different datasets. As shown in Figure 5B, MeL-STPhos_1 achieved the ACC, MCC, Sn, Sp and AUC values of 0.826, 0.652, 0.809, 0.842 and 0.904, respectively. Notably, MeL-STPhos_1 outperformed the top five baseline models on ACC, MCC and AUC values, with improvements ranging from 1.74% to 3.97%, 3.49% to 7.89% and 1.92% to 4.49%, respectively. On the A549 dataset, MeL-STPhos_2 achieved MCC, ACC, Sn, Sp and AUC values of 0.673, 0.836, 0.845, 0.828 and 0.913, respectively (Figure 5D). Specifically, MeL-STPhos_2 improved ACC, MCC and AUC by 2.13–4.08%, 4.26–8.15% and 1.84–3.34% compared to the top five baseline models. On the Vero E6 dataset, MeL-STPhos_3 achieved ACC, MCC, Sn, Sp and AUC values of 0.862, 0.725, 0.854, 0.870 and 0.930, respectively (Figure 5F). Its MCC, ACC and AUC values were 4.92% to 8.72%, 2.47% to 4.37% and 2.89% to 4.29% higher than those of the top five baseline models. Overall, MeL-STPhos consistently outperformed the top five baseline models on both training and independent datasets, demonstrating its high reliability, convergence and generalization capabilities.

### Performance comparison of MeL-STPhos and the existing predictors on the A549 dataset

Firstly, we compared the training performance between DeepIPs and MeL-STPhos_2 because they were trained/developed on the same dataset. Hence, the comparison is more straightforward and the result is shown in Table **S1** available online at http://bib.oxfordjournals.org/. Compared to DeepIPs, MeL-STPhos_2 showed improvements of 3.80, 5.60 and 2.40% in MCC, ACC and AUC values, respectively. These results demonstrate that the meta-learning approach, achieved through systematic analysis, significantly enhanced performance compared to the existing method on the training dataset.

Secondly, we evaluated the performance of MeL-STPhos on the A549 independent dataset and compared it to that of several established predictors, including MusiteDeep2017, MusiteDeep2020, DeepPSP and DeepIPs. As shown in Table 1, MeL-STPhos_2 achieved the best performance, with MeL-STPhos_1 coming in second place in terms of global metrics such as ACC, MCC and AUC values. Notably, MeL-STPhos_2 outperformed existing predictors by 2.69–3.47% in ACC, 4.13–6.94% in MCC and 1.95–3.70% in AUC. MeL-STPhos_2’s superior performance is attributed to its training and evaluation of identical cell-specific datasets. MeL-STPhos_1, trained on a mixture of cell lines, remained competitive with the best model. Unfortunately, MeL-STPhos_3 achieved the lowest performance, likely because a model trained on Vero E6 cells cannot be transferred to other cell lines. Notably, MusiteDeep2017, MusiteDeep2020 and DeepPSP maintained reasonable performance levels despite being trained on general phosphorylation site data, highlighting the applicability of models developed on generalized phosphorylation data for identifying phosphorylation from virus-induced cell-specific data. Overall, MeL-STPhos_2 significantly outperformed DeepIPs on both datasets, demonstrating the importance of our systematic approach to exploiting different feature descriptors, classifiers and meta-learning approach, which is responsible for such improved performance.

**Table 1:** Comparison of prediction performance with the most advanced methods by independent testing on the A549 dataset

Residue type	Method	ACC	Sn	Sp	MCC	AUC
S/T	MeL-STPhos_1	0.830	0.804	0.856	0.661	0.912
	MeL-STPhos_2	0.836	0.845	0.828	0.673	0.913
	MeL-STPhos_3	0.701	0.629	0.772	0.405	0.763
	DeepIPs	0.806	0.796	0.835	0.632	0.894
	DeepPSP	0.802	0.767	0.838	0.606	0.876
	MusiteDeep2020	0.810	0.830	0.790	0.620	0.887
	MusiteDeep2017	0.802	0.789	0.815	0.604	0.880

### Cross-model validation

**Figure 6:**
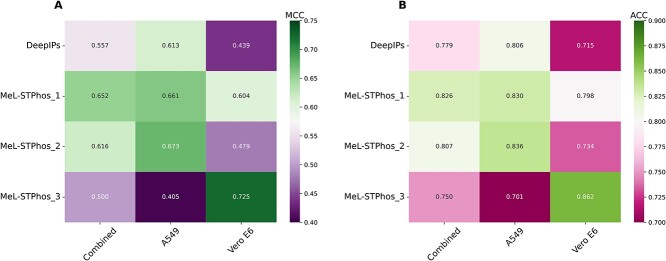
Cross-model validation performance on the three independent datasets, measured by (**A**) MCC and (**B**) accuracy (ACC).

We conducted a cross-model validation to assess the transferability of the cell-specific model to other datasets (only an independent dataset was used). This assessment aimed to identify the predictive model with the highest advantage in real-world scenarios. Figure 6 shows that MeL-STPhos_1 achieved excellent performance on three different datasets, with ACC over 80%. MeL-STPhos_2 performed similarly on its own and the combined datasets (ACCs of 0.836 versus 0.807), but its performance dropped significantly when tested on the Vero E6 cell line. As expected, the cell-specific models (MeL-STPhos_2 and MeL-STPhos_3) performed best on their datasets. Interestingly, MeL-STPhos_1 was better than the other models at identifying S/T phosphorylation sites in a variety of cell lines, including A549 and Vero E6 cells.

Next, we evaluated the transferability of our three models to Y phosphorylation site modification using the whole 102 positive samples and 102 negative samples from the DeepIPs dataset. Here, we excluded DeepIPs from the comparison because its S/T phosphorylation site prediction model is not applicable to other phosphorylation site prediction tasks. Figure S4 available online at http://bib.oxfordjournals.org/ shows that MeL-STPhos_2 achieved the best performance with MCC, ACC, Sn and Sp of 0.635, 0.814, 0.735 and 0.892, respectively. MeL-STPhos_1 achieved a reasonable performance, but its ACC was significantly lower than MeL-STPhos_2 (~10%). MeL-STPhos_3 achieved a random performance, indicating that it is not suitable for predicting other phosphorylation sites. When the Y phosphorylation sites from A549 cells were evaluated with the A549 S/T-specific model, MeL-STPhos_2, the model accurately predicted those sites. This suggests that a cell-specific model developed for S/T phosphorylation can be transferred to Y phosphorylation site prediction on the same cell because the surrounding 16 upstream and downstream residues may share a similar pattern.

### Feature contribution analysis

**Figure 7:**
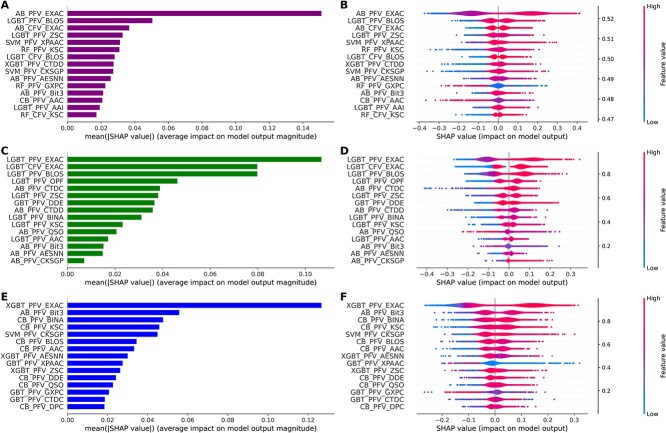
Average absolute SHapley Additive exPlanation (SHAP) values for crucial features, where larger values denote a more significant impact on model outcomes. (**A**), (**C**) and (**E**) correspond to the combined, A549 and Vero E6 datasets, respectively. SHAP values for the probabilistic feature vectors/class label feature vectors (PFV/CFV) are presented for MeL-STPhos. Feature values are color coded, with blue indicating low values and red representing high values. Positive and negative SHAP values reveal feature directionality in data-specific phosphorylation site prediction. Positive SHAP values correspond to predictions of phosphorylation sites, while negative values denote predictions of non-phosphorylation sites. (**B**), (**D**) and (**F**) correspond to the combined, A549 and Vero E6 datasets, respectively.

We performed a SHapley Additive exPlanations (SHAP) analysis on the optimal models derived from three distinct datasets. Figure 7 illustrates the mean SHAP values and their corresponding impact on model output. The mean SHAP values facilitate feature ranking based on their significance in the model, enabling a better understanding of the primary features that influence model predictions. This information can be used to interpret the model. However, the mean SHAP values do not reveal the direction of the impact (positive or negative). To elucidate directionality, we generated SHAP violin plots and used red and blue to represent the phosphorylation (positive) and non-phosphorylation site (negative) impacts of each feature, respectively, on the model prediction. Wider regions in the red area signify that the feature exhibits a more robust positive impact on numerous instances, while broader regions in the blue area denote a more significant negative impact on those instances.

Figure 7 shows the contribution of the top 15 features to the final prediction of MeL-STPhos_1, MeL-STPhos_2 and MeL-STPhos_3. Of the 22 diverse feature descriptors employed in this study, 12 descriptors (EXAC, BLOS, ZSC, XPAAC, KSC, CTDD, CKSGP, AESNN, GXPC, Bit3, AAC and AAI)-based baseline models, 14 descriptors (EXAC, BLOS, OPF, CTDC, ZSC, DDE, CTDD, BINA, KSC, QSO, AAC, Bit3, AESNN and CKSGP)-based baseline models and 15 descriptors (EXAC, Bit3, BINA, KSC, CKSGP, BLOS, AAC, AESNN, XPAAC, ZSC, DDE, QSO, GXPC, CTDC and DPC)-based baseline models respectively contributed to the final predictions of MeL-STPhos_1, MeL-STPhos_2 and MeL-STPhos_3. Among these descriptors, eight descriptors overlapped (AAC, AESNN, Bit3, BLOS, CKSGP, EXAC, KSC and ZSC) between these three models. Remarkably, EXAC, BLOS and ZSC emerged as the top three ranked features for MeL-STPhos_1; EXAC, BLOS and OPF emerged as the top three ranked features for MeL-STPhos_2; and EXAC, Bit3 and BINA were the top three ranked features for MeL-STPhos_3, playing a pivotal role, while the other features fulfilled a supplementary function. The most crucial features of MeL-STPhos_1, MeL-STPhos_2 and MeL-STPhos_3, as identified by SHAP analysis, correspond to the features generated by the best baseline models (AB-EXAC, LGBT-EXAC and XGBT-EXAC) for the combined, A549 and Vero E6 datasets, respectively. This highlights the importance of SHAP analysis in feature contribution and interpretation.

### Case study

**Figure 8:**
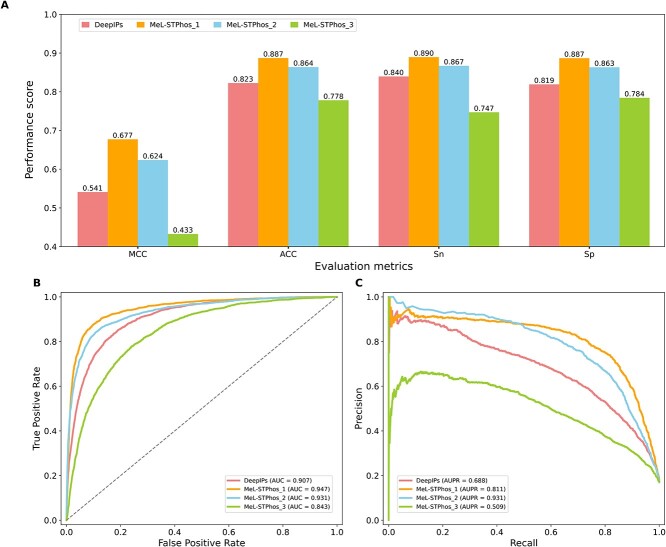
Performance comparison between MeL-STPhos and DeepIPs using the case study dataset. (**A**) Comparisons based on the MCC, ACC, sensitivity (Sn) and specificity (Sp) values; (**B**) the area under the receiver operating characteristic (ROC) curve (AUC); and (**C**) the area under the precision–recall curve (AUPR).

Recently, Valdes *et al*. [42] experimentally verified S/T phosphorylation sites in human IMR-90 cells that were infected with adenovirus type 2. To create a reliable dataset for a case study, we extracted phosphorylation and non-phosphorylation sites and excluded redundant sequences using a CD-HIT threshold of 70%; we thus obtained 2344 phosphorylation and 11 495 non-phosphorylation sites. This dataset provided a valuable opportunity to assess the potential applicability of the developed method to other viruses. Here, we compared the performances of our three models with the existing predictor DeepIPs. Results show that the MeL-STPhos_1 achieved the best performance, with MCC, Sn, Sp and ACC values of 0.677, 0.890, 0.887 and 0.887, respectively. Specifically, MeL-STPhos_1 improved MCC by 5.13%, 13.6% and 24.4% compared to the MeL-STPhos_2, DeepIPs and MeL-STPhos_3, respectively. This indicates that the generic model contains a sufficient amount of information to perform well when applied to other viruses. Note that MeL-STPhos_2 performed very well on its cell-specific data, but its performance dropped significantly when evaluated with other viruses, indicating that this model is suitable for its cell type, but not for other cell types. Figure 8B and C shows the performance comparison in terms of AUC and AUPR (area under the precision–recall curve), where MeL-STPhos_1 and MeL-STPhos_2 had the best performance in terms of AUC and AUPR, respectively. Based on Figure 8, MeL-STPhos_1 significantly outperformed the other two models proposed in this study and the existing predictor, indicating that MeL-STPhos_1 is the best available method for identifying phosphorylation sites of specific cells infected with viruses.

### Web server construction

To enhance accessibility for a wide range of users, we developed a dedicated web server, MeL-STPhos, which can be accessed at https://balalab-skku.org/MeL-STPhos. The web server was built using Django, Python, CSS, HTML and JavaScript, with a PostgreSQL database used for efficient storage and retrieval of job outcomes. The MeL-STPhos web server provides a comprehensive help page (https://balalab-skku.org/MeL-STPhos/help/), where users can find guidelines on how to use MeL-STPhos and download links to get the datasets used in the study. Users have two options for analysis: they can either upload a file containing multiple FASTA sequences or input one or more query sequences in FASTA format. After submitting the sequence(s), users must select the desired model (A549 or Vero E6 or Generic) and then proceed with job submission. If they do not select the desired model, the generic model (MeL-STPhos_1) will be used by default. Once a job is successfully completed, the results are presented on a dedicated interface, allowing users to easily view and analyze findings. In addition, users have the option to download the results in CSV format for future reference. If the users provide their email addresses when submitting the jobs, the result will be sent to their emails. To retrieve the outcomes of previous jobs, users can simply input the corresponding job ID into the ‘find job’ feature, conveniently located on the submission page. This feature enables users to access and review past job results with ease.

### Limitations and future studies

Although MeL-STPhos exhibited impressive performance in predicting phosphorylation sites across different datasets, some inherent limitations need to be addressed. To maintain consistent data distribution and facilitate the training of a robust and stable ML model, our study exclusively used data retrieved from the literature. This limitation could restrict a comprehensive understanding of phosphorylation sites. In future studies, we plan to expand the exploration of phosphorylation sites across different SARS-CoV-2 variants, including Alpha, Beta, Delta, Gamma and Omicron. We then aim to integrate the expanded data into our current computational framework or potentially develop deep learning-based methods [43–45]. Furthermore, we anticipate the construction of a cell-specific model based on future data. This strategy will pave the way for improving predictive accuracy and model application, ultimately leading to a more comprehensive and reliable understanding of phosphorylation sites.

## CONCLUSIONS

Identifying SARS-CoV-2 phosphorylation modification sites is a crucial step toward the development of new drugs and treatment approaches, indirectly benefiting global healthcare. Bioinformatics analysis has shown significant changes in protein phosphorylation events during infection of cells with SARS-CoV-2. However, the existing literature offers limited information on predicting phosphorylation modification sites related to observed changes. In this study, we addressed the limitations of word-embedding vectors by employing a multi-learning framework to produce improved protein sequence representations. The experimental outcomes indicate that MeL-STPhos can adaptively extract high-quality and discriminative features from various baseline models, leading to a substantial enhancement in prediction performance. Benchmark testing showed that MeL-STPhos_2 outperformed current methods on most evaluation metrics, further supporting the idea that protein sequences themselves hold enough information to predict SARS-CoV-2 phosphorylation modification sites. Cross-model analysis suggested that MeL-STPhos_1 is suitable for not only A549 and Vero E6 cells but possibly other cell types too, while MeL-STPhos_2 can be applied to identify Y phosphorylation sites. This also indicated that the S/T model can be applied to the Y dataset with great accuracy, laying the groundwork for future studies that combine different phosphorylation-site datasets and the development of a universal prediction model. To make this resource accessible to the relevant research community, we made the web server publicly available at https://balalab-skku.org/MeL-STPhos. Given the current scarcity of precise models for forecasting SARS-CoV-2 phosphorylation modification sites, our study presents an extensive approach that can serve as a foundation for subsequent investigations in this field. We expect that MeL-STPhos will prove a useful tool to complement wet laboratory experiments for identifying SARS-CoV-2 phosphorylation modification sites, aiding in the discovery of associated biological functions and facilitating a variety of sequence-oriented analyses.

**Key Points**

- Accurate identification of S/T phosphorylation sites in host cells infected with SARS-CoV-2 is vital for understanding protein-level changes and their functional roles in controlling cellular processes.
- We conducted a comprehensive assessment of 14 machine learning algorithms and 22 feature descriptors, leading to the development of a new meta-learning framework called MeL-STPhos.
- The meta-learning framework enhances protein sequence representations by overcoming the limitations of word-embedding vectors.
- Thorough cross-validation and independent tests demonstrated that MeL-STPhos outperforms current state-of-the-art tools.
- The MeL-STPhos web server provides a valuable resource for the broader research community and is freely accessible at https://balalab-skku.org/MeL-STPhos.

## Supplementary Material

final_supplementary_b_bbad433

## Data Availability

The datasets of MeL-STPhos are publicly available at: https://balalab-skku.org/MeL-STPhos/download/.

## Author Biographies

**Nhat Truong Pham** is a Ph.D. student at the Computational Biology and Bioinformatics Laboratory, Department of Integrative Biotechnology, Sungkyunkwan University, Republic of Korea. His research interests include artificial intelligence, bioinformatics, computational biology and medicine, deep learning, machine learning, signal processing, and XAI and optimization.

**Le Thi Phan** is a Ph.D. student at the Computational Biology and Bioinformatics Laboratory, Department of Integrative Biotechnology, Sungkyunkwan University, Republic of Korea. Her research interests include bioinformatics and data analytics in the fields of genome sequencing, drug discovery and immunity.

**Jimin Seo** is a graduate student in Young-Jun Jeon’s laboratory at Sungkyunkwan University, Republic of Korea. He is interested in developing bioinformatics and machine learning pipelines for identifying prognostic biomarkers for disease as well as characterizing chemo-resistance mechanisms in cancer cell biology.

**Yeonwoo Kim** is a graduate student in Young-Jun Jeon’s laboratory at Sungkyunkwan University, Republic of Korea. Her research interests include the development of bioinformatics pipelines and machine learning-based models for identifying prognostic biomarkers in solid tumors.

**Minkyung Song** is an assistant professor in the Department of Integrative Biotechnology, Sungkyunkwan University, Republic of Korea. Her research focuses on Bioinformatics and T-cell biology.

**Sukchan Lee** is a professor in the Department of Integrative Biotechnology, Sungkyunkwan University, Republic of Korea. He is a member of the Korea Society of Plant Pathology, Republic of Korea. His research interests include plant molecular virology, virus epidemiology, virus-resistant plant development and virus challenging model development.

**Young-Jun Jeon** is an assistant professor in the Department of Integrative Biotechnology, Sungkyunkwan University, Republic of Korea. The overarching goal of his research team is to identify biomarkers and drug-resistance mechanisms in cancer using cell biology and NGS-based approaches.

**Balachandran Manavalan** is an assistant professor in the Department of Integrative Biotechnology, Sungkyunkwan University, Republic of Korea. He is an associate member of the Korea Institute for Advanced Study, Republic of Korea. His research interests include artificial intelligence, bioinformatics, machine learning, big data and functional genomics.
