# Supplementary material for: Advancing the accuracy of SARS-CoV-2 phosphorylation site detection via meta-learning approach
Source: Brief Bioinform. 2023 Dec 6;25(1):bbad433. doi: 10.1093/bib/bbad433 (PMC10753650; doi:10.1093/bib/bbad433)
Supplement: final_supplementary_b_bbad433 [file final_supplementary_b_bbad433.pdf]

1. Methods for Bioinformatic Analysis

Principal Component Analysis (PCA): With the ggplot2 package (v.3.4.0) and DESeq2 package, we defined the function for PCA in R. We used the 500 genes with the largest variance across A549 cells infected with SARS-CoV-2 and uninfected cells at different hours post-infection.

Weighted Correlation Network Analysis (WGCNA): The traits are composed of Mock, SARS-CoV-2, and Hour and fed into the WGCNA package (v. 1.72-1) in R with gene expression of A549 and SARS-CoV-2 infected A549 [1]. The 'Hour' trait of samples was assigned to 4/6/9/24 values by hours after infection, respectively. The P-value cut-off (<0.1) remained 4 clusters enriched to the 'Mock' trait, and 6 clusters enriched to the 'SARS-CoV-2' trait. Remained mock enriched clusters are named 'Turquoise', 'Pink', 'Lightcyan', and 'Magenta', and SARS-CoV-2 enriched clusters are 'Purple', 'Tan', 'Lightgreen', 'Black', 'Green', and 'Red'.

Gene Ontology (GO) analysis: Pathway enrichment analysis of multiple lists of genes was conducted by Metascape online tool (v. 3.5) [2]. GO terms with a P-value < 0.01 were considered statistically significant. We enriched the gene lists into three main sets of GO terms, biological processes (BP), molecular functions (MF), and cellular components (CC). Each differentially regulated DEGs upon the samples at each infection time and the clusters 'Black', 'Green', and 'Red' and SARS-CoV-2 enriched clusters 'Lightcyan', and 'Magenta' were selected input gene lists, respectively. We excluded DEG lists of 24 hours-group because there were no enriched terms. Moreover, the pathway enrichment analysis was conducted with the in vivo lung tissue data (GSE190496)

Single sample Gene Enrichment Analysis (ssGSEA): ssGSEA was performed by applying the 255 overlapped GO terms found in DEG and WGCNA analyses to our normalized datasets. To compare the enriched score of the ssGSEA, we implemented the Wilcoxon-Rank sum test between the SARS-CoV-2-infected group and the uninfected group. We selected the commonly observed top 5 GO pathways in both SARS-CoV-2-

up-regulated DEGs (the results of DESeq2) and SARS-CoV-2-enriched clusters (the results of WGCNA), with the FDR < 0.1. We also selected the commonly observed top 15 GO pathways in both SARS-CoV-2-down-regulated DEGs and Mock-enriched clusters, with the FDR < 0.1.

2. Feature encodings

AAC: Amino acid composition (AAC) describes the proportions and arrangement of the 20 naturally occurring amino acids in a peptide sequence. Note that we considered a segment of a protein as a peptide sequence and described it accordingly. AAC offers valuable insights into the structural and functional properties of a peptide. AAC can be represented as a 20-dimensional (D) feature vector.

DPC: Dipeptide composition (DPC) characterizes the types and relative abundance of all possible consecutive amino acid pairs. A total of 400 possible dipeptides, each comprising two amino acids arranged in a specific order. DPC can be represented using the following equation:

$$\text{DPC} = (\text{number of dipeptides } AB \text{ / total number of dipeptides}) \quad (1)$$

where *AB* represents a specific dipeptide composed of amino acids *A* and *B*.

AAI: AAindex1 (AAI) version 9.2 is a comprehensive collection of 20 numerical values representing various physicochemical and biological properties of amino acids [3]. Encompassing 566 indices, with 553 free from NaN values, AAI provides a wealth of information for each amino acid within a sequence. We gathered these indices and subsequently averaged across the entire sequence.

The calculation of AAindex1 proceeds as follows:

$$AAI(j) = \frac{\sum_{L=1}^{N} AAI(AA_L)}{R} \quad (2)$$

where *j* represents the 553 AAIndex1 indices, AA_L denotes the amino acid at position *L*, and *R* signifies the total number of residues in the sequence.

CTD: Composition-Transition-Distribution (CTD) method comprehensively characterizes the amino acid properties of peptide sequences [4, 5]. This approach utilizes three distinct feature descriptors: composition, transition, and distribution. The composition descriptor (CTDC) measures the frequency of occurrence of each amino acid property group within

the sequence. In contrast, transition descriptor (CTDT) measures the frequency of transitions between polar and neutral groups of a specific nature. The distribution descriptor (CTDD) measures the frequency of amino acids with a specific property in the first 25%, 50%, 75%, and 100% of the peptide sequence. Employing the CTD method, a set of 21 descriptors was generated for each amino acid property. These features were calculated independently using 13 distinct physicochemical properties, yielding 39-D feature descriptors for CTDC and CTDT, and 195-D feature descriptor for CTDD.

CKSGP: Composition of k-spaced amino acid group pairs (CKSGP) method calculates the frequency of amino acid pairs separated by varying distances (*k* residues), where *k* can take on values from 0 to 10. Unlike the conventional *k*-spaced amino acid pair composition, CKSGP categorizes these pairs into five distinct groups based on the physicochemical properties of their constituent amino acids. Additionally, CKSGP classifies the DPC's into 25 distinct classes based on their properties. This procedure generates a total of 25 descriptors for each amino acid pair, encompassing all possible residue distance from 0 to 10. consequently, this method produces a 275-D feature vector.

KSC: *K*-spaced conjoint triad (KSC) method employs a comprehensive approach that entails grouping three peptides together, as detailed in [6]. The technique involves classifying all amino acids into seven categories and then considering every combination of three consecutive amino acids, resulting in a total of 343 unique features.

GXPC: Amino acids exhibit distinct physicochemical properties that can be categorized into five groups: aliphatic (IMGAVL), aromatic (FWY), positive charge (HKR), negative charge (EDG), and uncharged (QNSTCP). Leveraging these properties, the grouped DPC (GDPC) can be categorized into 25 classes, resulting in a 25-D vector. Similarly, the grouped tripeptide composition (GTPC) can be grouped into 125 categories, forming a 125-D vector. Finally, by combining the 25-D and 125-D through linear combination, a 150-D feature vector is obtained, termed GXPC.

XPAAC: It is a combination of pseudo AAC (PAAC) and amphiphilic PAAC (APAAC) to comprehensively represent peptide sequences. Similar to conventional AAC, PAAC captures local sequence information by encoding amino acid frequencies within a specific window size. On the other hand, APPAC captures global sequence information by considering the distribution of hydrophobic and hydrophilic amino acids along the peptide chain. Notably, both approaches employ the same parameters ($\lambda = 5$ and $w = 0.05$), resulting in a unified 55-D feature vector.

DDE: Dipeptide deviation from the expected mean (DDE) is a feature descriptor that emphasizes the fixed dipeptide composition, considering the codon diversity. Since amino acids are determined by combinations of three bases, the occurrence frequencies of dipeptides in sequences naturally vary. For a given sequence, DDE generates a 400-D feature vector by standardizing the directly calculated DPC using z-score normalization.

QSO: The Quasi-sequence-order (QSO) descriptor integrates local and global peptide sequence information by capturing both sequence-order effects and the physicochemical attributes of amino acids [7]. This process consists of four steps: (1) peptide sequence is divided into smaller subsequences or fragments, (2) for each amino acid in the subsequence, its physicochemical properties, such as hydrophobicity, polarity, and charge, are determined (3) identifying the sequence-order effect that signifies the spatial organization of amino acids within the peptide sequence, and (4) the physicochemical properties and sequence-order effect are combined to generate the QSO descriptor, resulting in a 100-D feature vector.

EGSM: This descriptor is a composite of four individuals: Shannon **E**ntropy, **G**eary autocorrelation, **S**equence-order-coupling number (SOCN), and **M**oran autocorrelation. The Shannon entropy for each sequence in the dataset is calculated using the following function:

$$H(X) = -\Sigma\ (P(x\_y) * \log2(P(x\_y))) \quad (3)$$

Here, *y* represents the 20 amino acids, and P(x_y) indicates the probability of a specific amino acid occurring within the sequence.

Both Geary's and Moran's autocorrelation descriptors are respectively constructed based on the arrangement and distribution of amino acid properties, originating from AAIndex1, along the sequence. Prior to calculating these descriptors, all indices are standardized. SOCN is derived from the distance matrix that encompasses the 20 amino acids. Two distance matrices are employed, based on the work of Grantham [8] and Schneider-Wrede [9], respectively.

ABC: It quantifies the combined total of atomic and bond compositions (ABC) for each amino acid sequence. This descriptor captures both the elemental composition and the bonding patterns. The atomic characteristics of a peptide sequence refer to the relative abundance of five elements (C, H, N, O, and S), while the bond characteristics include total bonds, single bonds, and double bonds.

AESNN: Alignment-based encoding of secondary structure and networks (AESNN) that represents each amino acid residue using a 3-D vector. These vectors are derived from the outputs of three hidden units in a neural network that has been trained on structural alignments, as reported in previous study [10, 11]. For a given peptide sequence, AESNN generates a 99-D feature vector.

Bit3: In binary 3-bit (Bit3) representation, a protein sequence is encoded using nonoverlapping 3-letter amino acid group {a1, a2, a3}. Each group is represented by a 3-D binary vector, and subsequently by a 5-D binary vector.
For instance, a1 is encoded as (100), a2 as (010), and a3 as (001). Amino acid categorization based on their physicochemical properties, such as hydrophobicity. The variation among subtypes 1 through 7 lies in the varying divisions of amino acids. Subtypes 2 to 7 employ a more detailed classification based on factors like "Secondary structure," "Normalized van der Waals volume," "Polarizability," "Polarity," "Charge," and "Solvent accessibility," as detailed in a previous study [12]. Combining all types generates a 693-D feature vector.

Bit5: Binary 5 bit (Bit5) is a combination of type 1 and type 2. In the first approach, peptide sequences are represented through a collection of five amino acid groups, labeled as {a1, a2, a3, a4, a5}. Each group is associated with a unique 5-D binary vector (*a1*∈{S, T, C, P, N, Q}, *a2*∈{F, Y, W}, *a3*∈{R, K, H}, *a4*∈{D, E}, *a5*∈{G, A, V, L, M, I}). These groups are subsequently denoted by binary vectors, with a1 as (10000), a2 as (01000), and so on, culminating in a5 as (00001). The second approach, type 2, delves into the numerous possible arrangements of ones and zeros within a five-bit unit. There are 32 potential configurations to represent the 20 amino acids. By discarding configurations that either contain no ones, all ones, or those with either 1 or 4 ones, we obtained 20 representations. The linear combination of these two methodologies, resulting in a 330-D feature vector.

OPF: The overlapping property features (OPF) descriptor represents amino acid sequences by systematically grouping amino acids into seven categories. The OPF descriptor encompasses three distinct subtypes, each arising from diverse amino acid classifications. The variations between type 1 and 3 subtypes arising from the specific groupings of amino acids. By combining these individual subtypes reuslting in the formation of 693-D feature vector.

BINA: Binary profile feature (BINA) method represents peptide sequences using binary code, where each amino acid is represented by unique sequence of 0s and 1s. This binary code effectively indicates the presence or absence of each of the 20 naturally occurring amino acids. Eventually, BPF encodes a 660-D feature vector for a given peptide sequence.

ZSC: Z-scale (ZSC) descriptor utilizes five distinct physicochemical properties to encode amino acid sequence. These properties, initially proposed by Sandberg and colleagues in 1998 [13], effectively capture the amino acid characteristics. This encoding method generates a 165-D feature vector.

BLOS: The BLOSUM62 matrix, frequently used in the BLAST sequence alignment program, serves as a toll for evaluating the similarity between two protein sequences. This matrix is widely employed to analyze the sequence conservation of related proteins

within large databases and has been utilized as a feature in various prediction models. With each of the 20 amino acids represented by a single row in the BLOSUM62 matrix, protein sequences can be encoded based on this matrix. BLOS generates a feature vector with 660-D (20 x 33) for a given peptide sequence.

EXAC: EXAC descriptor combines two approaches: enhanced AAC (EAAC) and enhanced grouped AAC (EGAAC). EAAC determines the AAC with a constant sequence window, while EGAAC uses grouped amino acid properties, as mentioned in GXAAC, within fixed-length windows. The resulting EXAC descriptor generates a 725-D feature vector.

Reason for excluding 2- and 12-hours samples

The initial dataset encompasses six distinct time intervals: 2, 4, 6, 9, 12, and 24 hours. We present a summary of the GSE18536 dataset in Figure 1. It illustrates that the samples taken at the excluded time points (2 and 12 hours) demonstrate a marked reduction in both the raw count of gene expressions and the overall gene diversity (61 and 0) when compared to samples from other time intervals. Given this significant reduction in gene diversity and raw count at these two specific time points, we excluded the 2-hour and 12-hour samples from our further analysis. This was done to focus on the other time points that provided a more comprehensive and informative view of gene expressions.

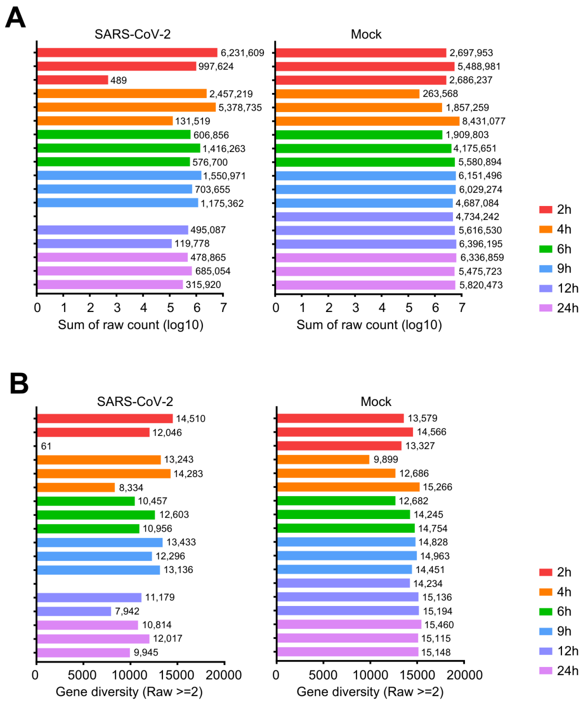

Figure 1. Basic quality control summary of the GSE184536. **(A) Sum of raw count:** It was calculated for each time point (2, 4, 6, 9, 12, and 24 hours) and expressed as a log10 value. This shows the total number of genes expressed at each time point. **(B) Gene diversity:** It was calculated by adding all genes with a count of 2 or greater at each time point.

Supplemental tables and figures

Table S1. Performance comparison of MeL-STPhos and the existing tool for S/T phosphorylation site prediction on the A549 training dataset.

Residue type	Method	ACC	Sn	Sp	MCC	AUC
S/T	MeL-STPhos_2	0.844	0.850	0.833	0.688	0.918
	DeepIPs	0.806	0.796	0.835	0.632	0.894

Figure S1. In vivo validation of the signatures in SARS-CoV-2 infected lung tissues. **(A)** PCA analysis showing the separation of the samples between normal and the infected samples, and **(B)** Volcano plot showing Differentially Expressed Genes (DEGs).

A

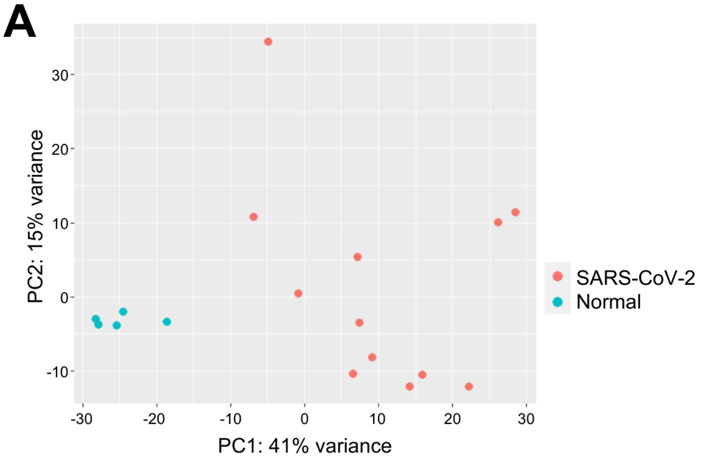

B

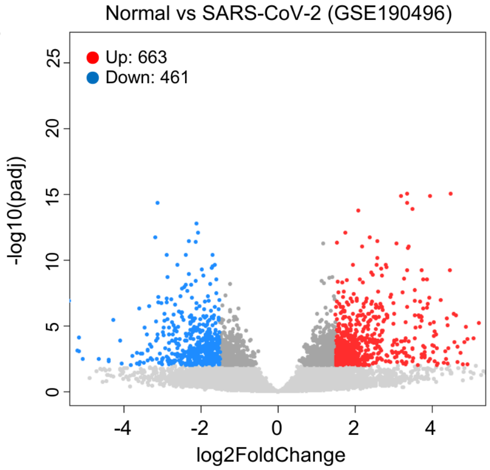

Figure S2. The average performances of each classifier over 22 different feature descriptors for the combined, A549, and Vero E6 datasets is depicted in panels **(A)**, **(B)**, and **(C)**, respectively.

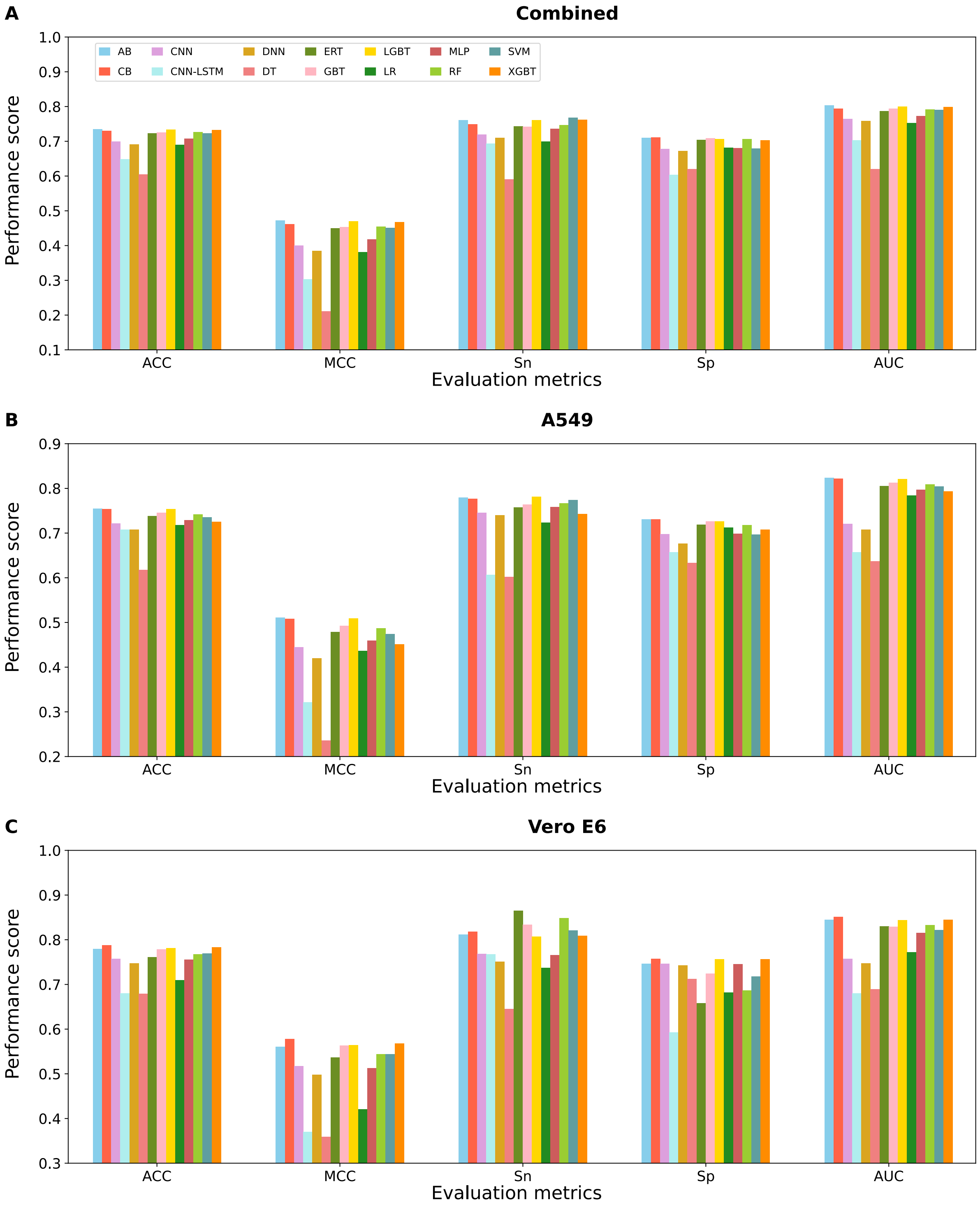

Figure S3. The performances of top five classifiers based on probabilistic feature vector (PFV), class label feature vector (CFV), and a combination of PFV and CFV (PCFV) for the combined, A549, and Vero E6 datasets, respectively.

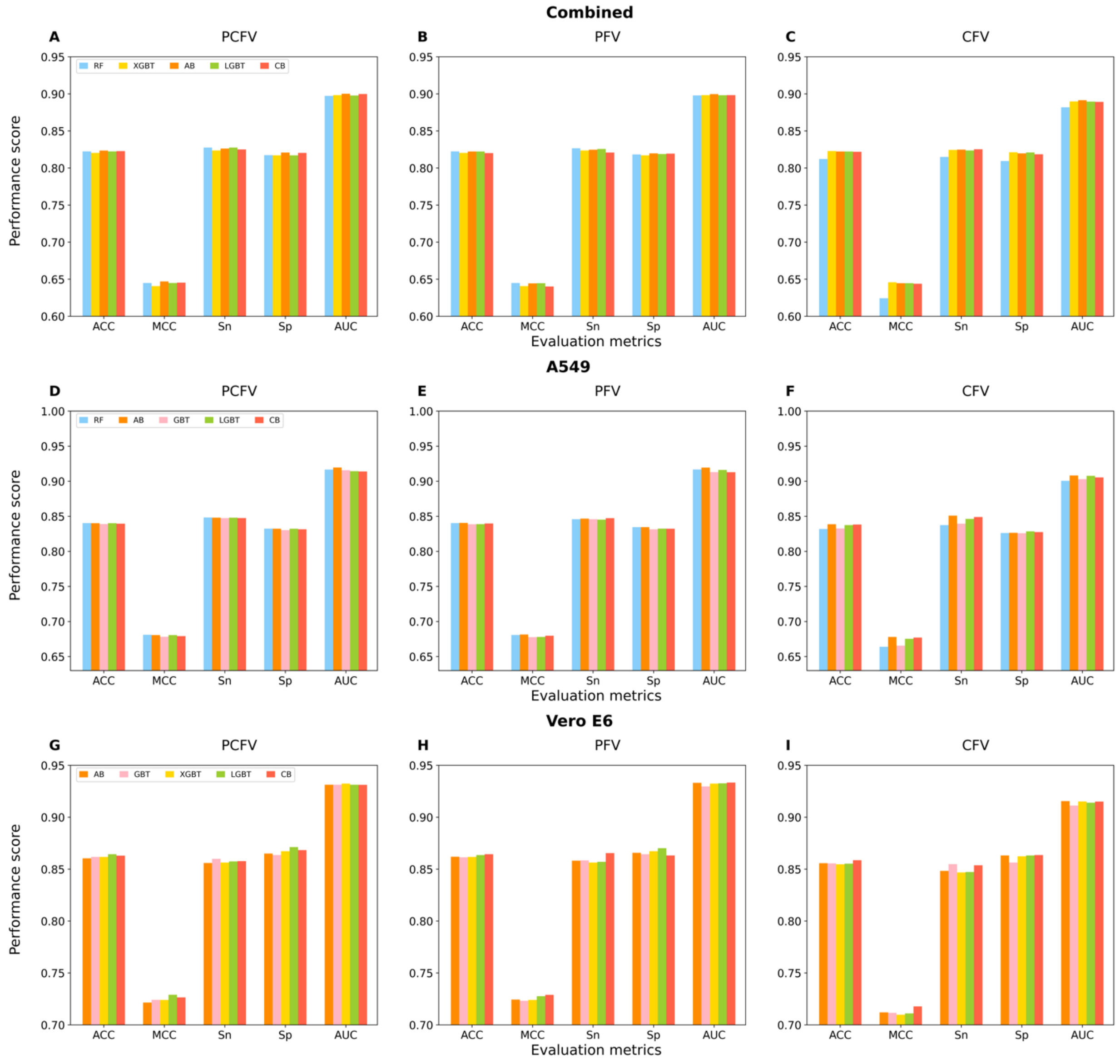

Figure S4. Cross-model validation performance using the whole positive and negative samples of tyrosine (Y) phosphorylation site modification in the A549 dataset. **(A)** Comparisons based on Matthews correlation coefficient (MCC), accuracy (ACC), sensitivity (Sn), and specificity (Sp) values; **(B)** the Area under the Receiver Operating Characteristic (ROC) curve (AUC); and **(C)** the Area under the Precision–Recall curve (AUPR).

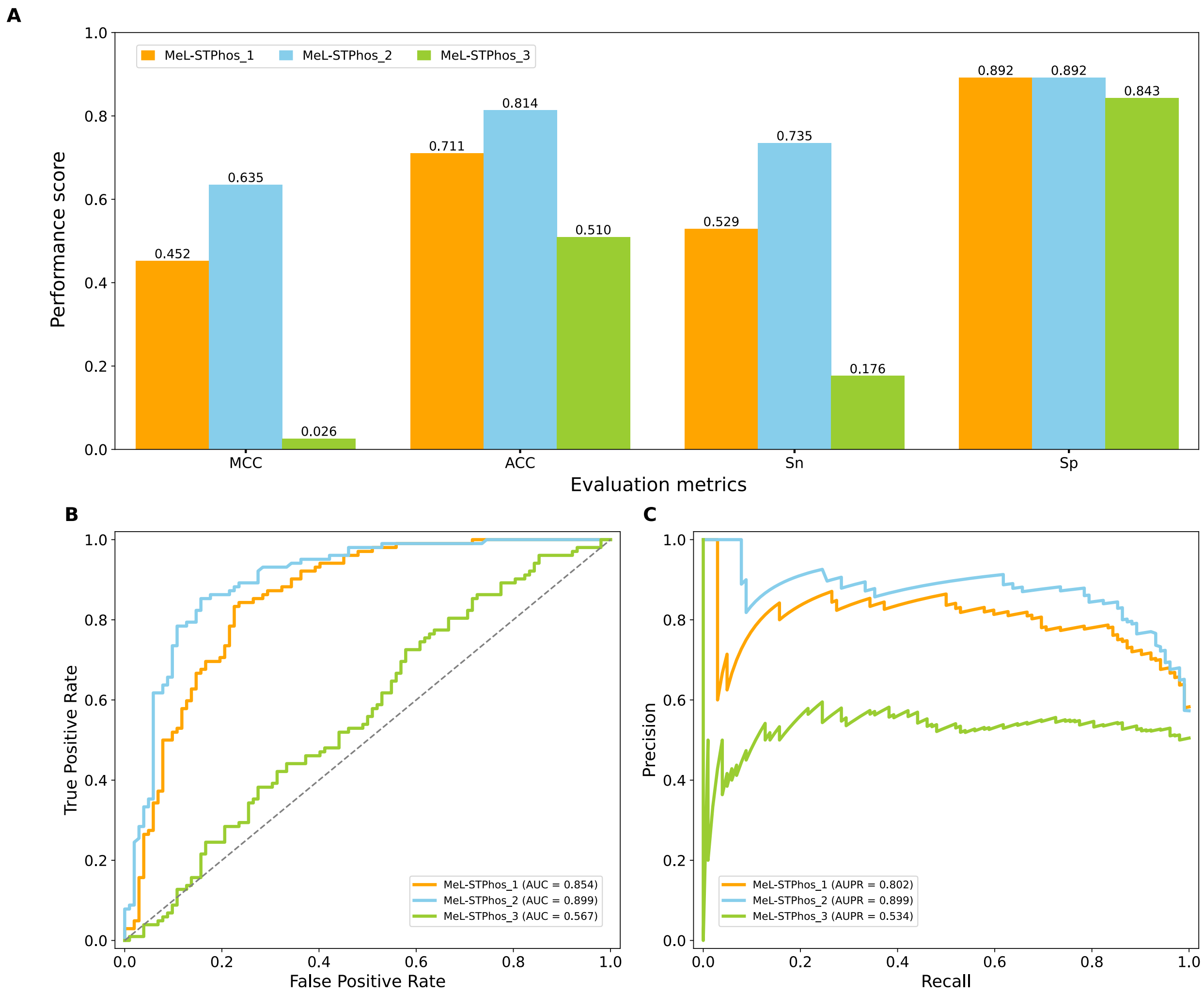

References

1. Langfelder P, Horvath S. WGCNA: an R package for weighted correlation network analysis, BMC Bioinformatics 2008;9:559.
2. Zhou Y, Zhou B, Pache L et al. Metascape provides a biologist-oriented resource for the analysis of systems-level datasets, Nat Commun 2019;10:1523.
3. Kawashima S, Pokarowski P, Pokarowska M et al. AAindex: amino acid index database, progress report 2008, Nucleic Acids Res 2008;36:D202-205.
4. Dubchak I, Muchnik I, Holbrook SR et al. Prediction of protein folding class using global description of amino acid sequence, Proc Natl Acad Sci U S A 1995;92:8700-8704.
5. Dubchak I, Muchnik I, Mayor C et al. Recognition of a protein fold in the context of the Structural Classification of Proteins (SCOP) classification, Proteins 1999;35:401-407.
6. Shen J, Zhang J, Luo X et al. Predicting protein-protein interactions based only on sequences information, Proc Natl Acad Sci U S A 2007;104:4337-4341.
7. Chou KC. Prediction of protein subcellular locations by incorporating quasi-sequence-order effect, Biochem Biophys Res Commun 2000;278:477-483.
8. Grantham R. Amino acid difference formula to help explain protein evolution, Science 1974;185:862-864.
9. Schneider G, Wrede P. The rational design of amino acid sequences by artificial neural networks and simulated molecular evolution: de novo design of an idealized leader peptidase cleavage site, Biophys J 1994;66:335-344.
10. Lin K, May AC, Taylor WR. Amino acid encoding schemes from protein structure alignments: multi-dimensional vectors to describe residue types, J Theor Biol 2002;216:361-365.
11. Liu B, Gao X, Zhang H. BioSeq-Analysis2.0: an updated platform for analyzing DNA, RNA and protein sequences at sequence level and residue level based on machine learning approaches, Nucleic Acids Res 2019;47:e127.
12. Chen Z, Liu X, Zhao P et al. iFeatureOmega: an integrative platform for engineering, visualization and analysis of features from molecular sequences, structural and ligand data sets, Nucleic Acids Res 2022;50:W434-W447.
13. Sandberg M, Eriksson L, Jonsson J et al. New chemical descriptors relevant for the design of biologically active peptides. A multivariate characterization of 87 amino acids, J Med Chem 1998;41:2481-2491.
